# Depth-Variable Settlement Patterns and Predation Influence on Newly Settled Reef Fishes (*Haemulon* spp., Haemulidae)

**DOI:** 10.1371/journal.pone.0050897

**Published:** 2012-12-14

**Authors:** Lance K. B. Jordan, Kenyon C. Lindeman, Richard E. Spieler

**Affiliations:** 1 Oceanographic Center, National Coral Reef Institute, Nova Southeastern University, Dania Beach, Florida, United States of America; 2 Department of Education and Interdisciplinary Studies, Florida Institute of Technology, Melbourne, Florida, United States of America; National Institute of Water & Atmospheric Research, New Zealand

## Abstract

During early demersal ontogeny, many marine fishes display complex habitat-use patterns. Grunts of the speciose genus *Haemulon* are among the most abundant fishes on western North Atlantic coral reefs, with most species settling to shallow habitats (≤12 m). To gain understanding into cross-shelf distributional patterns exhibited by newly settled stages of grunts (<2 cm total length), we examined: 1) depth-specific distributions of congeners at settlement among sites at 8 m, 12 m, and 21 m, and 2) depth-variable predation pressure on newly settled individuals (species pooled). Of the six species identified from collections of newly settled specimens (n = 2125), *Haemulon aurolineatum* (tomtate), *H. flavolineatum* (French grunt), and *H. striatum* (striped grunt) comprised 98% of the total abundance; with the first two species present at all sites. Prevalence of *H. aurolineatum* and *H. flavolineatum* decreased substantially from the 8-m site to the two deeper sites. In contrast, *H. striatum* was absent from the 8-m site and exhibited its highest frequency at the 21-m site. Comparison of newly settled grunt delta density for all species on caged (predator exclusion) and control artificial reefs at the shallowest site (8-m) revealed no difference, while the 12-m and 21-m sites exhibited significantly greater delta densities on the caged treatment. This result, along with significantly higher abundances of co-occurring piscivorous fishes at the deeper sites, indicated lower predation pressure at the 8-m site. This study suggests habitat-use patterns of newly settled stages of some coral reef fishes that undergo ontogenetic shifts are a function of depth-variable predation pressure while, for at least one deeper-water species, proximity to adult habitat appears to be an important factor affecting settlement distribution.

## Introduction

For many coastal fish species, larval settlement occurs in shallow areas spatially separated from those typically occupied by adult conspecifics, with species of grunts (Haemulidae) and their predators (Lutjanidae) as frequent examples [1], [2]. The process of utilizing multiple habitats during early life-history stages is commonly referred to as an ontogenetic habitat shift [3]. Individuals settling to shallow areas may be exposed to lower mortality and increased growth rates, with subsequent recruitment to offshore adult populations representing an important source of replenishment [4], [5]. Refuge from predation affects mortality on settlement stages of coral reef fishes, influencing adult population sizes and altering overall community structure [6]–[9]. It has been suggested that nearshore, back-reef habitats such as seagrass beds and mangroves support high densities of new settlers by providing size-appropriate refuge and increased prey availability (e.g., [10]).

Grunts of the genus *Haemulon* (15 western Atlantic species) can represent a major component of many Greater Caribbean coral reef fish communities and support important fisheries throughout the region [11]–[16]. A wide array of studies have focused on settlement or early juvenile habitat use in grunts (studies include but are not limited to [2], [6], [17]–[30]). Morphological and ecological transitions among larval, early settlement, and early juvenile life-history stages of grunts are more complicated than in many other reef fish families [31], [32]. Grunts settle earlier than many other reef fish genera in terms of both size [23], [33] and age [18], [34]. Before the acquisition of species-specific stripe and caudal spot pigment patterns of early juveniles (approximately 2 to 5 cm in length), species-level identifications typically require microscopic examination using meristic, morphometric, and pigment characters [32]. Many grunts can form high-density, multispecies schools at settlement, which can reduce mortality [35], [36] but identification challenges have often limited species-level ecological research on settlement-stage individuals.

Year-round settlement of some prominent grunt species occurs to reef and other shallow natural habitats (e.g., seagrass beds, mangroves, hardbottom, patch reefs, rubble zones, and reef pavements) [6], [23], [28], [37]–[40]. Additionally, artificial reefs (ARs) in the wider Caribbean can support high densities of early-stage and juvenile grunts [20], [28], [41]–[46]. In southeast mainland Florida, newly settled grunts were recorded at substantially higher densities on concrete ARs than on a nearby reef which had the highest densities of any natural reef habitat in the area [47], [48]. Consistent with results of other studies that recorded early juvenile and juvenile stages of many grunt species in shallow-water habitats [23], [49], [50], examination of data from the continuous natural reef tracts throughout Broward County, Florida revealed that the abundance of early-stage (<5 cm SL) individuals was limited to depths <12 m [15], [28]. However, on ARs (including vessel reefs and small [∼1 m^3^] experimental units), high densities of early-stage grunts have been recorded at 21 m depth [46], [51]. The presence of early-stage grunts at depths >12 m suggests the use of shallow habitats at settlement is not obligatory for some species of *Haemulon*, especially when artificial structures are present.

Adult population densities of reef fishes may vary with distance from settlement and juvenile habitats. For several *Haemulon* species in the Caribbean, reefs in close proximity to mangroves and seagrass beds exhibited higher adult abundances than reefs spatially isolated from recruit source habitats [52]. Comparisons of new settler and juvenile densities have also revealed that many species utilize back-reef habitats rather than windward coral reefs typically occupied by the adult reproductive population segment [30]. Lower predation rates on back-reef habitats utilized by early-stage fishes could, in part, explain the cross-shelf, age-structured distributional patterns observed in many species [21], [53]–[55]. However, in most studies that compare predation among different habitat types, experimental designs were not able to address potentially confounding factors associated with among-habitat variations in depth, distance to the reef, topographic complexity, benthic fauna, and other biophysical factors; all of which can influence predator-prey interactions.

Several studies have examined how reef fish assemblages respond to differences in predation pressure and topographic complexity using ARs [41], [45], [56]. Fish abundance and species richness may correlate to reef area/volume, rugosity, isolation distance, and elevation, while variations in benthic fauna influence fish assemblage structure of reefs [48], [57]–[60]. Experimental manipulations that use ARs to remove or reduce the confounding effects of these factors allow much sharper focus on specific processes. To investigate potential factors responsible for the cross-shelf distributions of newly settled stages of *Haemulon* species in southeast Florida, we examined 1) settlement patterns at the species-level using ARs deployed at three discrete depths and 2) depth-specific differences in relative predation pressure on new settlers by comparing caged versus control AR treatments at each depth, while simultaneously measuring the abundance of co-occurring predators.

## Materials and Methods

### Ethics Statement

Sampling was conducted under Florida Fish and Wildlife Conservation Commission Special Activity License 06SR-982 and 06SR-978.

### Study Area and Sites

**Figure 1:**
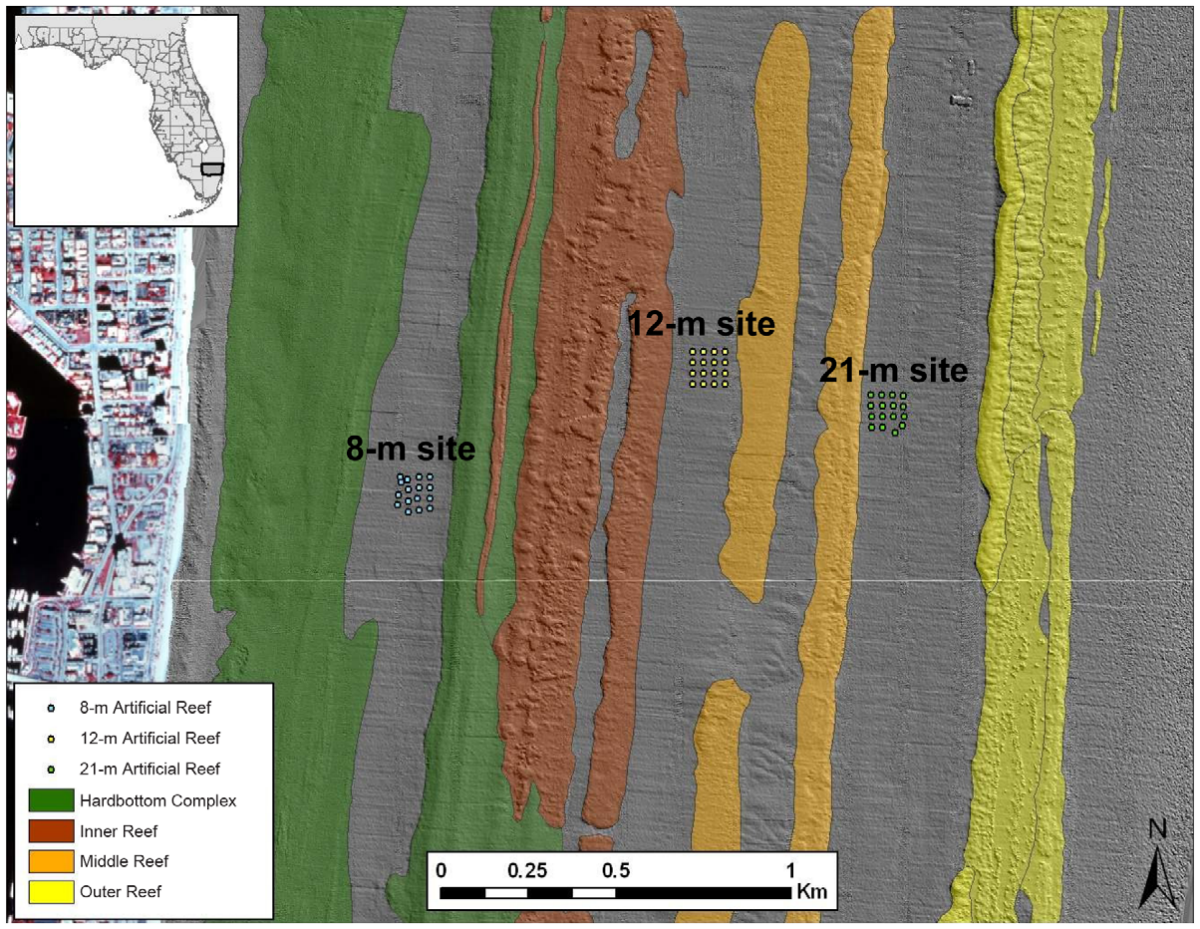
Reef categorization (based on LIDAR data) of the study area (Broward County, Florida, USA) and site locations of artificial reefs. Sand habitat in gray.

In order to minimize variability of habitat structure and associated ecological processes among depth treatments, three sites located on sand plains between nearly continuous reef tracts that parallel the shore of Broward County, Florida, USA were chosen [61]. The study sites lie at water depths of 8 m, 12 m, and 21 m (Fig. 1) and were located at almost the same latitude (8-m: 26°07.4 N, 80°05.8 W; 12-m: 26°07.6 N, 80°05.3 W; and 21-m: 26°07.5 N, 80°04.9 W). At each of the three sites, a 4×4 grid of ARs was chosen in which ARs were separated by approximately 30 m. The 30-m spacing was selected based on previous studies in the area which showed this distance adequately minimized movements of resident fishes among ARs and was short enough that, given typical horizontal visibility for the area, divers could efficiently navigate the grid using compass headings [45], [48]. All replicate ARs were >30 m from any natural reef structure. Sandy plain habitats in the area are generally flat and homogenous in terms of structure.

### Replicate Artificial Reefs

**Figure 2:**
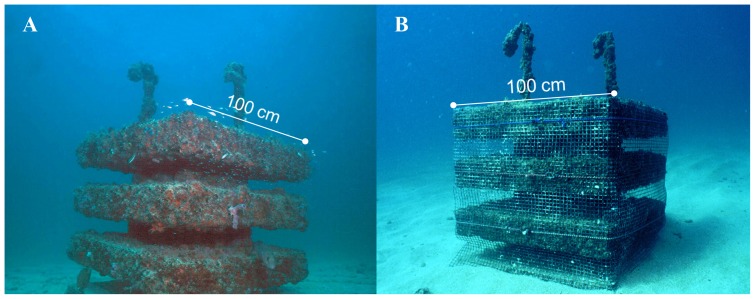
Replicate artificial reefs treatments A) noncaged [NC] control and B) caged [C] to exclude predators.

Forty-eight replicate ARs (Gilliam-Spieler reefs; *sensu* [28]) at three sites (16 each) were used in the present study (Fig. 2A). The ARs were ∼1 m^3^ (L×W×H; 100 cm×100 cm×96 cm) cubes constructed using concrete block amalgamated with cement and reinforced with steel rods (rebar), weighing ∼1.5 tons prior to deployment. Each AR contained four layers separated by nine support columns. The void space, rugosity, and overhangs created by the design of the ARs are used by newly settled grunts in addition to other species. The flat, vertical sides of the ARs used in this study allowed for fastening of plastic netting material to exclude larger fishes (potential early-stage *Haemulon* predators) from the internal structure (Fig. 2B). All ARs were initially deployed in early 1995 and remained submerged.

Eight of the 16 ARs, at each of the three sites, were randomly chosen for the caged treatment during each experimental trial. For the caged treatment, four ∼1 m^2^ sections of 1.9 cm (3/4″) polyethylene mesh netting were fastened to vertical sides of an AR to limit piscivore access. Two bungee cords secured the netting around the top and bottom layers of each of the four vertical sides of the AR. The vertical corner edges of the netting were joined together using cable ties. Prior to the start of the experiment, large encrusting organisms (e.g., oysters, bryozoans, etc.) were removed from the vertical surfaces of ARs so the netting would fit directly against the vertical sides without gaps.

### Study Design and Data Collection

Previous examination of temporal settlement patterns of grunts in this area showed highest settlement in summer months [28]. Based on McFarland et al. (1985) [23], experimental trials bracketed the quarter moon phases (waxing and waning) to ensure data collections captured pulses of newly settled stages of grunts. Visual surveys and specimen collections were performed every two weeks during the summer of 2006. To start the study, all fishes were removed from the ARs using rotenone. The initial clearing of fishes, and effective start of the study, took three dive days: 8–10 May 2006. The first data collection date was 22 May 2006. The final collections were conducted on 10–11 August 2006. For each experimental trial used in the analysis, all counts and collections at each site were performed within a two- to four-day period (Table 1).

**Table 1:** Sampling dates of each site during respective experimental trials.

Experimental Trial	8-m	12-m	21-m
Initial Fish Clearing	5/9/06	5/10/06	5/8/06
Trial #1	5/23/06	5/24/06	5/22/06
Trial #2	6/6/06	6/7-8/06^a^	6/5/06
Trial #3	6/22/06	6/21/06	6/19/06
Trial #4^b^	N/A	N/A	7/10/06
Trial #5^c^	7/25/06	7/26/10	7/24/06
Trial #6	8/11/06	8/11/06	8/10/06
Trial #7^d^	8/21/06	8/21/06	8/21/06

a Required two days to conduct visual counts and collect specimens due to vessel engine failure.

b Schedule for three weeks to account for shifting lunar phase. Rough seas did not allow for data collection at 8-m and 12-m sites. Data from 21-site used for length comparisons only.

c No visual count data was obtained. Rotenone was applied to clear fishes for subsequent experimental trial.

d Trial exhibited low NS grunt abundance (and occurrence) values and was used only for species distributional analysis.

In terms of multiple criteria, transitions among early-life stages of grunts are more complicated than in many reef fish families [31]. Post-flexion larvae (5–10 mm standard length, SL) can often be epibenthic with an extended demersal metamorphosis showing morphological and ecological attributes of larvae (e.g., planktivory) and, following Lindeman and Richards (2006) [32], we considered newly settled (NS) individuals to be those from 5–20 mm SL (5–10 mm: epibenthic larvae; 10–20 mm transitional new settler). In the current study, assuming direct settlement from the plankton (not secondary settlement from other habitat), the maximum age of specimens able to settle onto the ARs was theoretically limited by the number of days (14) between experimental trials. At 40 days, length is approximately 18 mm SL in H. *flavolineatum* [18] and other *Haemulon* species (Lindeman, unpubl. data).

Abundance of grunts was recorded using visual counts of individual ARs. All grunts located within a one-meter radius of an AR were recorded to the nearest cm total length (TL). A similar visual census method has been used in several other studies on ARs [42], [45], [48], [62]. In addition to recording abundance of grunts, divers also recorded the abundance of species that may prey upon *Haemulon* individuals (i.e., Holocentridae, Serranidae, Apogonidae, Carangidae, Lutjanidae, and Scorpaenidae) present within one meter radius of an AR. Visual counts were not time delimited.

To examine depth-variable predation intensity of NS grunts, two treatments at each of the three sites were used. The first treatment was represented by ARs lacking the outer plastic netting on the vertical sides, hereafter the noncaged (NC) treatment. This treatment represented the control for the other treatment type, the caged (C) treatment. Plastic netting was secured to the vertical sides of the ARs of this treatment type as described above. For every experimental trial, the treatment type at each site was randomly assigned to the individual ARs to control for any influence caused by AR position within the grid. At each site, half (eight) of the 16 ARs were assigned as C while the other half were NC. Use of these two treatments allowed for relative comparisons in predation intensity among sites. A significantly higher mean delta density (see below) of NS grunts on the C treatment relative to the NC treatment at a given site (i.e., depth) was considered to have a higher relative predation pressure than a site lacking significant difference between the two treatments. The randomized-block experimental design used in this study was meant to provide independence between successive experimental trials and an acceptable mode of interspersing the replicates; to avoid pseudoreplication [63], [64].

Connell (1997) [65] suggested that a partially caged treatment could be used as an adequate control in assessment of predation impacts. Using the same AR design and caging material as the present study, Gilliam (1999) [45] assessed attraction of fishes to caged ARs by comparing a partially caged treatment (netting on two sides) to full caging and noncaged treatments (the latter two treatments were identical to those used in the present study). The results of that study showed that partial caging (predator accessible) and fully caged (predator exclusion) treatments did not significantly differ for 0–2 cm TL fishes (all species; *Haemulon* spp. represented the large majority, >95%); these treatments exhibited significantly higher abundances than the noncaged treatment. This implies the effect of the caging material on the abundance (and potential attraction) of newly settled fishes was not proportional to the amount of caging material.

After completing a visual count of an AR, NS grunts were collected by herding individuals into fine-mesh hand nets. Approximately 80% of the individuals recorded during visual counts were collected. After collection, rotenone was applied to clear fishes from the AR, establishing the effective start of the subsequent experimental trial. In addition to the grunts, apogonids (resident predators) were also affected by the piscicide. Approximately 300 g of rotenone powder (7.4%) was placed into a re-sealable plastic bag with approximately 240 mL of Ivory™ liquid dish soap. One bag was used for each AR. Fishes associated with the AR were enveloped in the rotenone cloud and most fishes died within five minutes. Once cleared of fishes, each AR was assigned its randomly predetermined treatment (caged or noncaged) for the subsequent fortnightly experimental trial and divers moved on to the next AR. Upon returning to the boat, collected specimens were placed into labeled jars with 90% EtOH for preservation.

For newly settled *Haemulon* individuals, *in situ* species-level identification when total length was <2 cm TL was usually not possible. Species identification for collected specimens was performed in the laboratory using Lindeman and Richards (2006) [32]. Once identified to species, specimens were measured to the nearest 0.01 mm standard length (SL). Because some collection samples contained thousands of specimens, subsampling techniques were used. For each experimental trial, samples from four ARs from each of the three sites (depths) were randomly chosen for species identification and length measurement. Collections from two caged and two noncaged ARs were represented in the four selected samples from each site. Individual samples were then subsampled volumetrically using a Folsom splitter. A subsample containing approximately 80 specimens was used to represent the species composition and length frequency of the raw sample.

### Statistical Analysis

Previous studies on both natural reef and ARs have shown that NS stages of grunts exhibit a spatially and temporally patchy distribution [45], [47], [48]. Frequency-distribution histograms (not shown) revealed highly right-skewed abundance data in which zero values were common. This patchy distributional pattern is common in visual fish surveys and can cause extremely high variability among replicate samples with corresponding mean abundances exhibiting statistically high variances [66], [67] in which parametric statistics are unlikely to appropriately resolve among-factor differences. Thus, NS *Haemulon* spp. abundance data collected in visual counts were analyzed using the delta approach [68], [69].

To calculate delta density of newly settled grunts for each factor, all zero data were removed from raw abundances (hereafter, concentration; conc). A frequency-distribution histogram was constructed which showed that the data were still highly right (positively) skewed. A log_10_(x+1) transformation was applied to meet the assumptions of analysis of variance (ANOVA). If a significant difference (p<0.05) was detected, a modified Tukey HSD test (for unequal sample size) was performed to determine differences among variables. Percent presence (i.e., occurrence; occ) data were also calculated for each factor; either NS *Haemulon* were present on an AR or they were not. To compare occurrence data among the three sites and between treatments at each site (pooling all experimental trials) a Kruskal-Wallis nonparametric ANOVA was performed. If significant (p<0.05), a nonparametric multiple comparisons test was run to identify among-site differences. A Mann-Whitney U test was used to compare occurrence data of C and NC treatments overall (pooling all experimental trials and sites). A Fisher's exact test (two-tailed p-value) was used to corroborate factor differences identified in nonparametric multiple comparisons test (from Kruskal-Wallis) and Mann-Whitney tests.

Delta density is a composite density represented by the product of occurrence (the proportion of zero to non-zero values) and concentration (mean abundance after removal of all zeros). For each site, treatment, and site/treatment combination delta density (D) was calculated enabling comparison between C and NC replicate reefs at each site. The product of concentration (conc) and occurrence (occ) was calculated for each site, treatment, and site/treatment combination to yield indices of relative density represented by delta density. It has been suggested that delta density is a better representation than conventionally calculated mean density because the latter can have a large variance due to the presence of zero values [70]. Statistical comparisons of delta density would not be possible without an error term. Estimated delta density variance (var) can be calculated using a Taylor approximation [71]. Approximated delta density variance was calculated through the product of the square of occurrence and the variance of concentration:
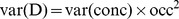
Once the estimated variances (and standard deviations) for delta densities were calculated, it was possible to statistically compare delta density between pairs of sites, treatments, and treatments at each site using a difference test (t-test). This was achieved by using “difference between two means” test (Statistica, Statsoft, Inc.) in which means (represented by delta density of a factor) and corresponding approximated standard deviations were entered to calculate a two-tailed p-value.

Additionally, the abundance of predatory fishes (based on visual census of individual ARs) was compared using a three-way ANOVA with experimental trial, sites, treatments as factors. A log_10_(x+1) transformation was applied to raw abundance data to meet the assumptions of analysis of variance (ANOVA). If a significant difference (p<0.05) was detected, a Tukey HSD *post hoc* analysis was used to identify the differences among variables.

## Results

### Species-Specific Depth Patterns at Settlement

A total of 2125 newly settled (NS) *Haemulon* species (<20 mm SL) collected from ARs was identified to the species level. This subsample represented ∼24% of the total abundance of NS grunts recorded from the visual counts of the ARs. Six species were collected: *Haemulon aurolineatum* (tomtate), *H. flavolineatum* (French grunt), *H. striatum* (striped grunt), *H. melanurum* (cottonwick), *H. parra* (sailors choice), and *H. plumierii* (white grunt). Only the first three species were abundant enough to warrant further examination of depth/site settlement distribution.

**Figure 3:**
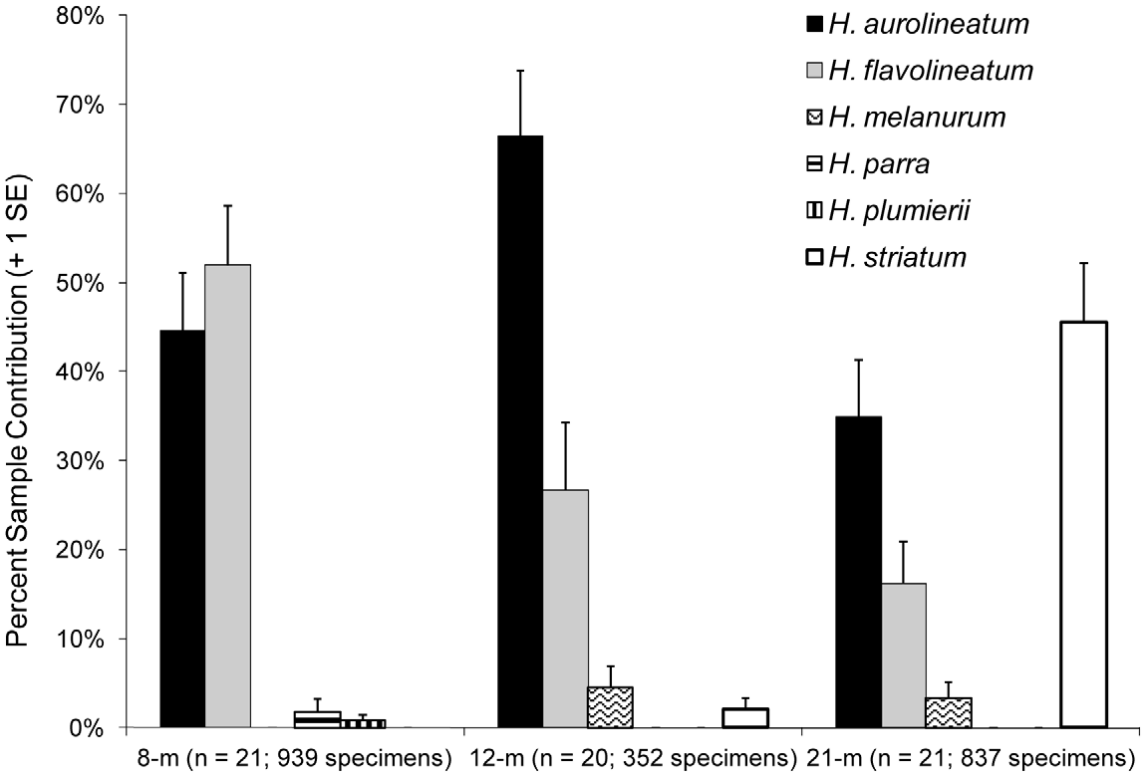
Mean percent sample contribution for newly settled stages of each *Haemulon* species. Data pooled for experimental trials and treatments.

Data for individual species, standardized by sample size (mean species contribution per sample at each site, pooling all experimental trials), revealed *H. aurolineatum* as the predominate species collected in this study (Fig. 3). At the 8-m site, *H. aurolineatum* ([mean ± SE] 44.6±6.5) and *H. flavolineatum* (52.0±6.7) exhibited similar percent contributions (per sample). However, mean percent contribution for *H. aurolineatum* was two times greater than *H. flavolineatum* at the 12-m and 21-m sites. At the 21-m site, *H. aurolineatum* exhibited a mean percent sample contribution of approximately 35%. Although 98% of NS *H. striatum* were collected at the 21-m site, its mean percent contribution only accounted for 45.6% (±0.07) of the samples collected at this site.

**Figure 4:**
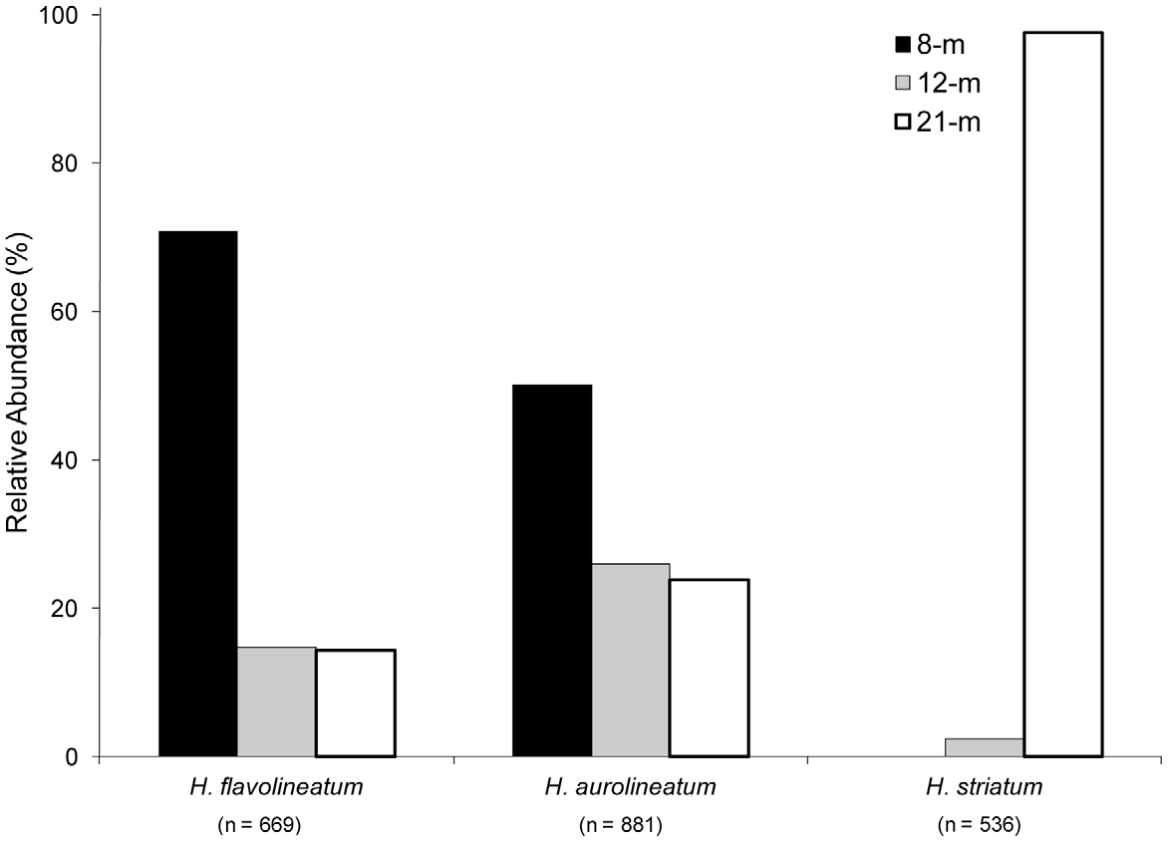
Relative abundance of species of *Haemulon* at each site, based on specimen totals from collections of new settlers on ARs (pooling all experimental trials).

*Haemulon aurolineatum* and *H. flavolineatum* were found at all three sites and exhibited depth-related differences in their distributions (Fig. 4). Of the *H. aurolineatum* specimens collected, 49% were found at the 8-m site while 26% and 24% were recorded from samples at the 12-m and 21-m sites, respectively. Similarly, at the 8-m site, 71% of all *H. flavolineatum* specimens were collected. The remainder of specimens was collected at nearly equal relative abundance (∼14%) from the 12-m and 21-m sites. In contrast to these two species, *H. striatum* was collected on the 12-m and 21-m sites only; with ∼98% present on the deepest site. The remaining species were not abundant: NS stages of *H. parra* and *H. plumierii* were found exclusively at the 8-m site (13 and 3 individuals, respectively), while 18 NS *H. melanurum* were found at the 12-m and 21-m sites only.

### Among-Site Comparison of Settlement and Predation

#### Settlement of *Haemulon* Species

A total of 8842 newly settled grunts was included in the following analyses of visual count data. Grunts were patchily distributed among ARs resulting in heavily right-skewed abundance data, inappropriate for parametric statistics. Utilization of the delta approach required calculation of concentration and occurrence in order to obtain delta density values. A three-way ANOVA using concentration (i.e., abundance after removal of zeros; log_10_[x+1] transformed) of NS grunts revealed significant differences for experimental trial, treatment, and the site by treatment interaction term (Table 2). A Tukey HSD test revealed that the significant difference in concentration of NS grunts that occurred for the site×treatment interaction resulted from a difference between the 12-m NC replicate reefs and C replicate reefs at the 12-m and 21-m sites. None of the other site/treatment combinations differed from one another. A Tukey HSD test for unequal sample size also revealed more consistency in concentration of NS grunts among experimental trials than seen for abundance. The caged AR treatment exhibited a significantly greater concentration than noncaged ARs (pooling experimental trials and sites). Experimental trial #2 had a significantly lower concentration than experimental trials #1 and #3. Experimental trial #6 did not differ from any of the others (Table 2).

**Table 2:** Results from 3-way ANOVA on NS grunt concentration (i.e., abundance after removal of zeros; log_10_[x+1] transformed).

Source	df	SS	MS	*F*	p
Site – Depth	2	0.320	0.160	0.632	0.534
Treatment	1	2.582	2.582	10.210	0.002*
Experimental trial	3	3.275	1.092	4.317	0.007*
Experimental trial×Site	6	2.610	0.435	1.720	0.126
Experimental trial×Treatment	3	0.990	0.330	1.305	0.278
Site×Treatment	2	2.869	1.435	5.672	0.005*
Experimental trial×Site×Treatment	6	1.330	0.222	0.877	0.516

Due to low abundances, experimental trial #7 was excluded from this analysis. P-values with asterisk (*)indicate significant difference.

Analysis of occurrence (i.e., the percentage of ARs on which NS grunts were recorded) was performed using Kruskal-Wallis nonparametric ANOVAs and revealed significant differences among sites (df = 2, p = 0.003) and experimental trials (df = 3, p<0.0001). Comparison between the C and NC treatments (pooling experimental trials and sites) showed no significant overall difference in occurrence (Mann-Whitney U = 4224.0, p = 0.243). However, comparison between caged and noncaged ARs at each site revealed a significant higher occurrence on caged ARs at the 12-m site (U = 368.0, p = 0.024), with no difference detected between treatment at either the 8-m or 21-m sites. For sites, a *post hoc* multiple comparisons test revealed that the 8-m site exhibited a significantly higher occurrence than the 12-m site. Neither of these two sites differed from the 21-m site. A *post hoc* multiple comparisons test showed that experimental trial #3 had a significantly higher occurrence of NS grunts than experimental trial #6, with no differences among the remaining experimental trials.

**Figure 5:**
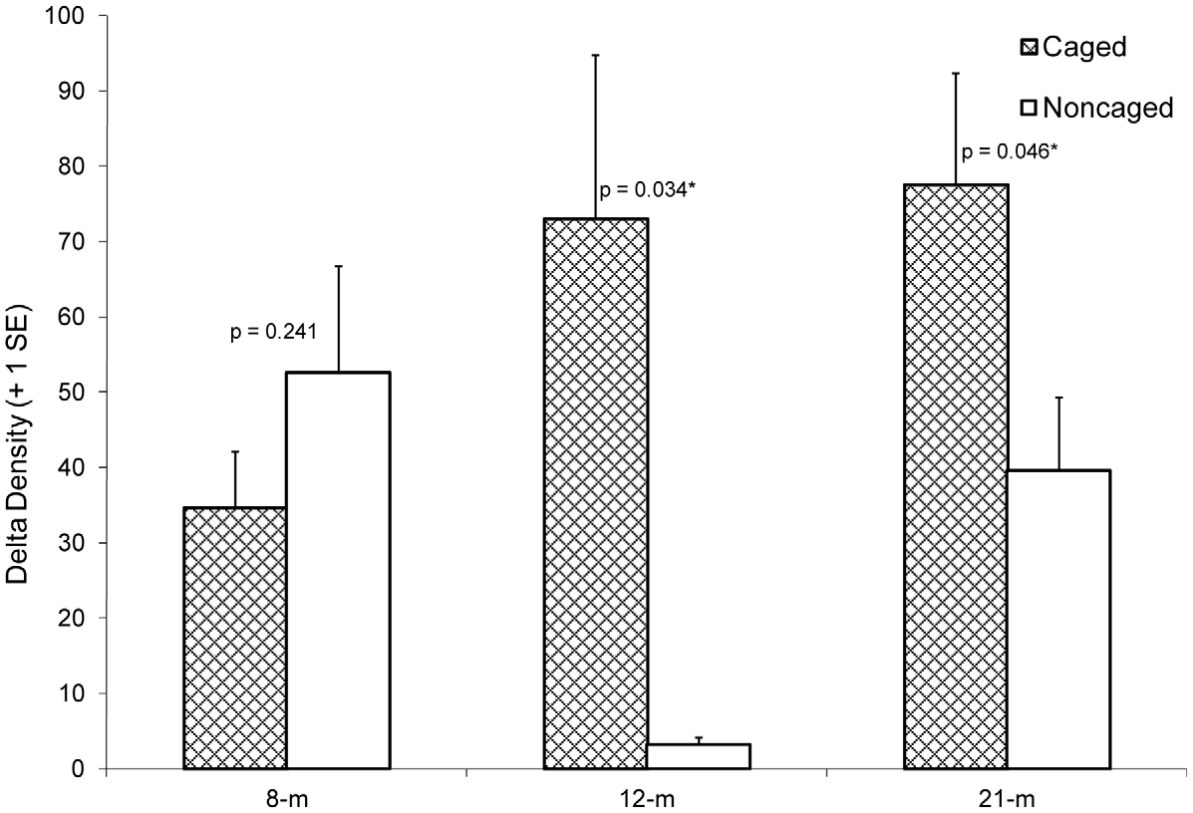
Mean delta density of newly settled stages of *Haemulon* species on the caged and noncaged ARs at each site. P-values obtained from comparison of caged and noncaged ARs for each site (difference between two means test). Significant p-values between treatments at a site denoted with asterisk (*).

No significant difference was found for delta density of NS grunts between any pair of sites (i.e., 8-m vs. 12-m, 8-m vs. 21-m, 12-m vs. 21-m). Comparison of delta densities for C (51.35±0.99) and NC (26.32±0.62) treatments (pooling data from all experimental trials and sites) revealed significantly higher values for the C treatment (p = 0.014). For treatment comparisons at each site, no significant difference was found between the treatments at the 8-m site (p = 0.241). However, the C treatment exhibited significantly higher delta densities than NC treatment at the 12-m (p = 0.034) and 21-m (p = 0.046) sites (Fig. 5).

#### Piscivore Distributions

Recording potential predators of NS grunts during visual counts at the ARs allowed for comparison of piscivore distributional trends across the shelf. Potential predators included species within several families: Holocentridae, Serranidae, Apogonidae, Carangidae, Lutjanidae, and Scorpaenidae. The five most abundant predators of NS grunts were: *Apogon pseudomaculatus* (twospot cardinalfish, Apogonidae), *Diplectrum formosum* (sand perch, Serranidae), *Caranx crysos* (blue runner, Carangidae), *Lutjanus analis* (mutton snapper, Lutjanidae), and *Cephalopholis cruentata* (graysby, Serranidae). A three-way ANOVA of piscivorous fish abundance (all species combined, log_10_[x+1] transformed) revealed a significant difference among experimental trials and sites (Table 3). A Tukey HSD test indicated mean piscivorous fish abundance was significantly lower on the 8-m site, with the other two depths exhibiting no difference (Table 4). No significant difference in overall piscivorous fish abundance between C and NC treatments (pooling all sites and experimental trials) was found (F = 0.47, p = 0.49).

**Table 3:** Results from 3-way ANOVA of piscivorous fish abundance (all species combined, log_10_[x+1] transformed).

Source	df	SS	MS	F	p
Site – Depth	2	10.059	5.030	46.764	<0.001*
Treatment	1	0.050	0.050	0.469	0.494
Experimental trial	4	1.459	0.365	3.391	0.010*
Experimental trial×Site	8	1.112	0.139	1.293	0.249
Experimental trial×Treatment	4	0.131	0.033	0.306	0.874
Site×Treatment	2	0.137	0.069	0.639	0.529
Experimental trial×Site×Treatment	8	1.013	0.127	1.178	0.314

Data from experimental trial #7 included. P-values with asterisk (*)indicate significant difference.

**Table 4:** Mean piscivore abundance (± SE) on ARs (pooling both treatments using data from all experimental trials) at the three study sites.

Species	8-m	12-m	21-m
*Apogon psuedomaculatus*	0.31±0.09^b^	3.04±0.53^a^	2.61±0.49^a^
*Diplectrum formosum*	0.50±0.10^b^	1.71±0.18^a^	0.28±0.09^b^
*Caranx crysos*	0.06±0.04^b^	1.16±0.44^a^	1.01±0.23^a^
*Lutjanus analis*	0.13±0.04	0.13±0.04	0.30±0.08
*Cephalopholis cruentata*	0^b^	0.03±0.02^b^	0.28±0.05^a^
Total*	1.34±0.19^b^	6.59±0.66^a^	5.33±0.63^a^

Differing letters indicate significant differences between sites using log-transformed data (log_10_[x+1]) (Tukey HSD, p<0.05).

* Based on all potential predators of newly settled stages of *Haemulon* species (see Methods).

For *A. pseudomaculatus*, the most abundant piscivorous species recorded, results of a three-way ANOVA mirrored the among-site abundance pattern (and Tukey HSD significance levels) exhibited by all piscivorous species combined (F_2_ = 20.64, p<0.001; Table 4). A three-way ANOVA of *D. formosum*, the most prevalent (i.e., highest occurrence and second-most abundant) piscivore recorded in this study also revealed a significant difference in abundance among sites (F_2_ = 56.14, p<0.001). However, for this species, a Tukey HSD test showed that its abundance was significantly higher at the 12-m site than the 8-m and 21-m sites, which did not differ from each other (Table 4).

A total of 180 *C. crysos*, a transient predator, was recorded in 47 of the 240 AR counts. On the 12-m and 21-m sites, *C. crysos* exhibited mean abundances greater than 1.0; significantly greater than the 8-m site (F_2_ = 9.18; p<0.001; Table 4). Large schools (containing hundreds of individuals) of *C. crysos* made passes by the ARs, frequently extended beyond the one-meter radius around the AR in which abundance data from visual surveys were recorded. It is likely that abundance (and percent presence) data collected during visual counts did not adequately represent the foraging behavior exhibited by this species.

## Discussion

### Depth-Specificity of Settlement among Congeners

Many reef fishes exhibit age-structured, cross-shelf distributional patterns whereby new settlers and juveniles utilize different habitat(s) than adult conspecifics [1], [72], [73]. Use of shallow habitats by new settlers implies that an advantage exists in differential habitat use. Several studies have demonstrated that utilization of shallow habitats by juvenile and newly settled individuals can reduce predation rates by providing size-appropriate structural complexity, which reduces predator foraging efficiency [54], [74]. Predation refuge provided by structural complexity may vary among species [75], [76] and may, in part, explain demographic differences in habitat-use patterns. Our results revealed varying settlement distributions among closely related species within the genus *Haemulon*.

Factors that influence depth and habitat specificity at settlement and the possible roles of predation remain poorly understood. Studies attempting to examine these factors are often confounded by within-habitat variation. In this study, collections and laboratory identification of 2125 newly settled individuals (i.e., epibenthic larvae) from ARs placed at similar sites at three depths (8 m, 12 m, and 21 m) indicated the most abundant species (*H. aurolineatum*, *H. flavolineatum*, and *H. striatum*) exhibited overlapping, yet distinct, distributional patterns. NS *H. aurolineatum* and *H. flavolineatum* were collected at all sites while *H. striatum* was collected at the 12-m and 21-m sites only (98% at the deepest site). Abundances of *H. aurolineatum* and *H. flavolineatum* were almost equal at the shallowest site, with the former species exhibiting comparatively higher abundance at the two deeper sites (Fig. 4).

The analogous depth distribution of settlement stage *H. aurolineatum* and *H. flavolineatum* seen in this study also reflects highly similar morphological adaptations, as new settlers and early juveniles share nearly identical pigmentation, eye size, and body shape [33]. Evidence suggests that all species of *Haemulon* feed on plankton during post-settlement stages [19], [32], [77]. Through ontogeny, individuals of *H. flavolineatum* become benthic carnivores as adults. However, juvenile and subadult *H. aurolineatum* often continue to feed diurnally on zooplankton while adults feed on both zooplankton and benthic prey [78]. The comparatively deep settlement of *H. striatum* likely reflects distinct ecological and morphological adaptations. This species is a highly specialized obligate planktivore throughout its life history, inhabiting the water column of deep reefs [78]–[80]. Settlement to relatively deep, offshore areas with more consistent availability of planktonic food resources could position individuals to grow faster, which can lower mortality rates [81]–[83]. Variability in food resources is unlikely to explain the species-specific distributional patterns within the genus since all species of *Haemulon* feed on plankton as new settlers [84]. For *H. flavolineatum* in Aruba and Curaçao, despite greater food abundance and corresponding faster growth rates of individuals exposed to offshore reef habitats, mangroves and seagrasses remained the predominant settlement habitats [29].

Because several species (which typically settle to shallow habitats) were capable of settlement on ARs in relatively deep waters, the possibility exists that the lack of NS grunts on deeper natural reefs >21 m [28] is indicative of 100% mortality at (or immediately following) settlement. However, the similar delta densities of new settlers (consisting of three primary species) on ARs at all three sites does not support this assumption. While differences in predation pressure among sites could be attributed to differences in predatory species composition and relative abundance, predation does not appear to be the only factor driving the distributional patterns of new settlers. Subsamples of collected specimens revealed that, while *H. aurolineatum* and *H. flavolineatum* settled at lower densities on the 21-m site than the 8-m site, *H. striatum* was never collected at the shallowest site. For *H. striatum*, settlement occurred at depths in closer proximity to adults or adult habitat, which may reduce mortality during subsequent habitat shifts. This implies that the ecological advantage gained by settling to deeper reefs (with higher pressure, see below) may outweigh the benefit of settlement to shallow habitats with potentially less predation pressure.

### Depth-Variable Predation Effects on the Cross-shelf Distribution of New Settlers

To gain understanding of how depth-variable post-settlement predation affects the distribution of newly settled individuals, the difference in NS grunts (all species) delta density between the C and NC treatments was used as a measure of relative predation pressure. The difference at each site was then compared among the 8-m, 12-m, and 21-m sites. While analysis revealed no among-depth difference in NS grunt delta density (see above), results of the caging experiment revealed higher relative predation pressure on NS grunts at the deeper sites. This pattern is likely to reflect conditions of the surrounding natural reef system. Larger (more consumptive) predators are likely to be more abundant in topographically complex (e.g., elevation, rugosity, volume, etc.) habitats [85].

In the reef system surrounding the study sites, several distinct hardbottom reef habitats are separated by sandy plains [61]. The nearshore ridge complex (NRC) habitat, adjacent to the 8-m site, exhibited the highest abundance of NS grunts relative to the other surrounding natural reef habitats (Jordan et al., in prep). Despite the high density of NS grunts, of the three reef habitat categories examined in Walker et al. (2009) [60], the NRC had lowest total fish abundance and species richness while also exhibiting lowest values of habitat elevation (m), volume (m^3^), surface rugosity index, and linear-rugosity index. Similarly, Almany (2004) [86] found that new settler abundance did not differ among reefs with varying topographic complexities. While size-appropriate refugia (topographic complexity, rugosity, etc.) for NS grunts may exist on deeper reefs in the area, the shelter characteristics of the shallow reefs may be less suitable for larger fishes and predators. The potentially size-appropriate refugia present in deeper reef habitats could simultaneously increase predation pressure on prey species [87].

Predation vulnerability has been shown to vary among prey species [88]. While this study was not designed to resolve variations in mortality among congeners, the different distributional patterns observed could be attributed to a species-specific response to predation pressure. Disproportionately higher predation pressure on newly settled *H. aurolineatum* and *H. flavolineatum* at the deeper sites would explain declining offshore abundances of these species. In contrast, *H. striatum* may have exhibited comparatively low mortality at the deepest site. Due to the underlying differences of the reef types, it is impossible to directly ascribe the findings of the caged-versus-noncaged treatment effects using ARs located at different depths to the surrounding natural reef system. Structural or biological attributes of the ARs absent from natural reefs (and *vice versa*) may allow *H. aurolineatum* and *H. flavolineatum*, normally associated with the shallow habitats, to settle to deeper areas. The structural complexity of the ARs used in this study (relative to natural reef) may have reduced priority effects and provided more size-appropriate refuge for newly settled individuals while negatively influencing the success rate of predatory strikes, relative to natural reef habitat [20], [86], [89]–[92]. Despite this difference, only the use of replicate artificial reef units (positioned on nearly identical habitat) could allow for an unconfounded examination of depth-variable predation pressure.

The mortality risk associated with undergoing ontogenetic habitat shifts are thought to outweigh the benefit gained by remaining in the settlement habitat [3]. The findings of Dahlgren and Eggleston (2000) [4] supported this assertion and suggested that ontogenetic habitat shifts minimize the ratio of mortality risk to growth rate. Such shifts in habitat use appear to provide a means of balancing predation (driven by appropriate refuge or predator abundance) and suitable food availability, thereby decreasing mortality. Habitat shifts, which offset the mortality risk of remaining within the former habitat, are likely to occur on a practical spatial scale. Results of our study suggest greater predation pressure on deeper reef areas and, in general, the length of time spent away (and distance travelled) from refuge is related to mortality risk [93]. Thus, the deep settlement of *H. striatum* appears to be driven by accessibility to habitat needed during subsequent life-history stages, which inhabit deep forereef areas to feed on relatively plankton-rich waters. The other two abundant species (*H. aurolineatum* and *H. flavolineatum*) shift to a benthic feeding mode at an early age (∼5 cm TL) and exhibited a broader settlement distribution that likely reflects the availability of habitats suitable for these species during subsequent life stages. Sandy areas, on which the conspecific adults often forage, are common throughout the reef tracts at all depths [61].

While results from this study suggest predation and proximity to adult conspecifics can influence new settler distribution on reefs, other factors may explain the observed patterns. Due to their common absence from plankton surveys, complex larval taxonomy, and low published abundance in light traps, understanding of species-specific *Haemulon* larval ecology is lacking [94], [95]. However, larval grunts may be able to detect and react to reef noise during settlement [96]. It is also possible that settling individuals avoid areas where they have detected resident predators/competitors, as seen with other species [92]. On the natural reef system surrounding the study area, small-bodied predators (serranids and apogonids) and territorial pomacentrids (*Stegastes* spp.) exhibited significantly lower abundances on shallow reef habitats (Jordan and Spieler, unpub. data; [15]), which opposed the distributional patterns seen for early-stage grunts [28]. Predator avoidance occurs among freshwater systems affects species distribution [97], [98]. In the case of settling larvae, predator avoidance behavior would require risk assessment of species that might negatively influence their survivorship [99]. Relative to other coral reef fishes, the lack of pelagic morphological features, small settlement size, and extended duration of the epibenthic larval period of grunts suggest that larvae often may not enter the pelagic realm, potentially staying in near-bottom association with softbottom habitat during much of the larval period [31], [32]. Such an early life history strategy could reduce planktonic mortality and explain the large schools of epibenthic larvae often found on the edges of shallow hardbottom structures for many species of *Haemulon*. As with many other reef fish families, research into sensory abilities of late-stage *Haemulon* larvae would be of value [100].

Although potential predators did not show higher abundances on the C treatment, which exhibited higher densities of newly settled individuals on the 12-m and 21-m sites, among-site differences in abundance were observed for *A. pseudomaculatus*, *D. formosum*, *C. crysos*, *C. cruentata*, and total piscivores (all species combined). Total potential piscivore abundance was significantly lower at the 8-m site. In general, all piscivorous species exhibited increasingly higher abundances on the offshore sites except *D. formosum* which occurred in 80% of the counts at the 12-m site, with only 35% and 20% at the 8-m and 21-m sites, respectively. Although the use of rotenone likely caused an unnatural feeding opportunity, this serranid was observed consuming early-stage grunts during rotenone sampling and has been shown to negatively affect fish recruitment on ARs [101]. The most abundant potential piscivore recorded, *A. pseudomaculatus*, was also observed feeding on NS grunts during collections. Marnane and Bellwood (2002) [102] showed that, despite their small size, fishes comprised a major dietary component of several species of Indo-Pacific apogonids. Similarly, *C. crysos* was observed feeding on NS grunts during collections, suggesting predator-prey interactions may also occur naturally. Although *C. crysos* abundance and its effect on NS grunt mortality was likely inadequately represented in the visual count data, studies have suggested carangids account for high mortality of new settlers on ARs in the Greater Caribbean [44], [48], [103]. Compared to other piscivores recorded in this study, its large size and schooling, chase behavior suggest that *C. crysos* would be a more consumptive predator [104]. However, the predation pressure placed upon prey NS grunts by this carangid was likely to be higher on noncaged ARs, since complete refuge from their predatory strikes could be obtained within the netting material. Thus, of the most prevalent piscivorous species recorded, it is possible that *C. crysos* may have contributed to the difference in NS grunt density between C and NC treatments at the 12-m and 21-m sites.

This study suggests that distributional patterns of NS grunts on the natural reef, in which the vast majority of individuals were recorded on nearshore habitats [28], are driven by multiple factors. Results from the comparison of C and NC treatments suggest that predation pressure was strongest at the deepest site. Although delta density of new settlers (all species) at this site did not differ from the other sites, the observed species-specific settlement patterns appear in part to reflect an ecological trade-off between predation pressure and proximity to adult conspecifics (or adult resources) for several *Haemulon* species. Depth does not appear to be a primary determinant of settlement for the two most prevalent species observed, *H. aurolineatum* and *H. flavolineatum*, which are typically opportunistic with regard to habitat selection at settlement [24]. The high settler and juvenile densities of certain ontogenetic-shifting species commonly found in shallow habitats may be the result of lower relative predation, corresponding to the density and constituents of the piscivore suite, rather than the increased structural refuge associated with certain habitats (e.g., seagrass, mangrove, etc.). However, for species within the genus *Haemulon*, distributional patterns at settlement do not appear to be driven solely by predation pressure. At settlement, all newly settled *Haemulon* species exhibit very similar morphologies and behaviors. Evidenced by its absence from shallow habitat, *H. striatum* may gain an ecological advantage by limiting the distance needed to shift from settlement to juvenile and adult habitats; offsetting the initial benefit of settling to shallow habitats with lower relative predation pressure.
